# Supplementary material for: Combinatorial prediction of marker panels from single‐cell transcriptomic data
Source: Mol Syst Biol. 2019 Oct 24;15(10):e9005. doi: 10.15252/msb.20199005 (PMC6811728; doi:10.15252/msb.20199005)
Supplement: Supplementary file 1 — Appendix [file MSB-15-e9005-s001.pdf]

Appendix Table of Contents

- Appendix Figure S1
    - Combinatorics overview
- Appendix Figure S2
    - COMET method overview
- Appendix Figure S3
    - 3-gene panel example
- Appendix Figure S4
    - XL-mHG test benchmarking
- Appendix Figure S5
    - XL-mHG test benchmarking on simulated Gaussian expression
- Appendix Figure S6
    - XL-mHG test benchmarking on a generative model
- Appendix Figure S7
    - Flow validation of T-cell markers
- Appendix Figure S8
    - B-cell staining for predicted marker combination
- Appendix Figure S9
    - Signature testing for B-cell sub-populations
- Appendix Figure S10
    - Validation for predicted marker combination on B-cell subpopulation
- Appendix Figure S11
    - Cloud deployment chart for COMET availability

Figure S1

A

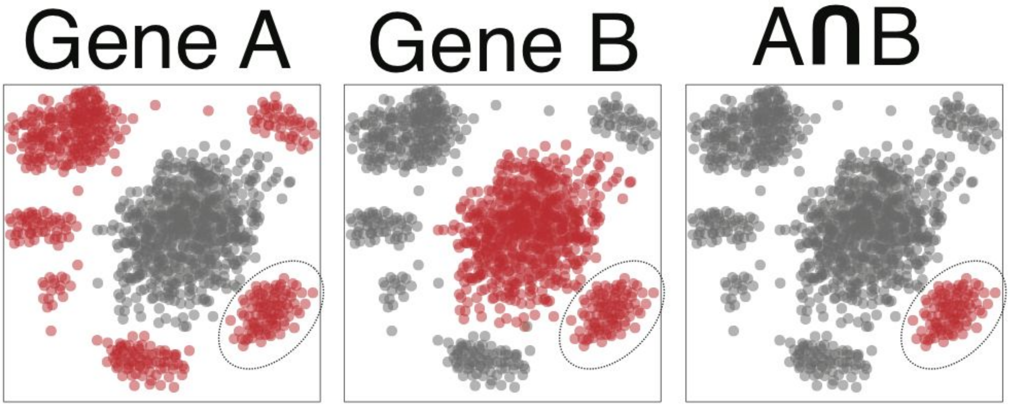
Gene A
Gene B
A∩B

B

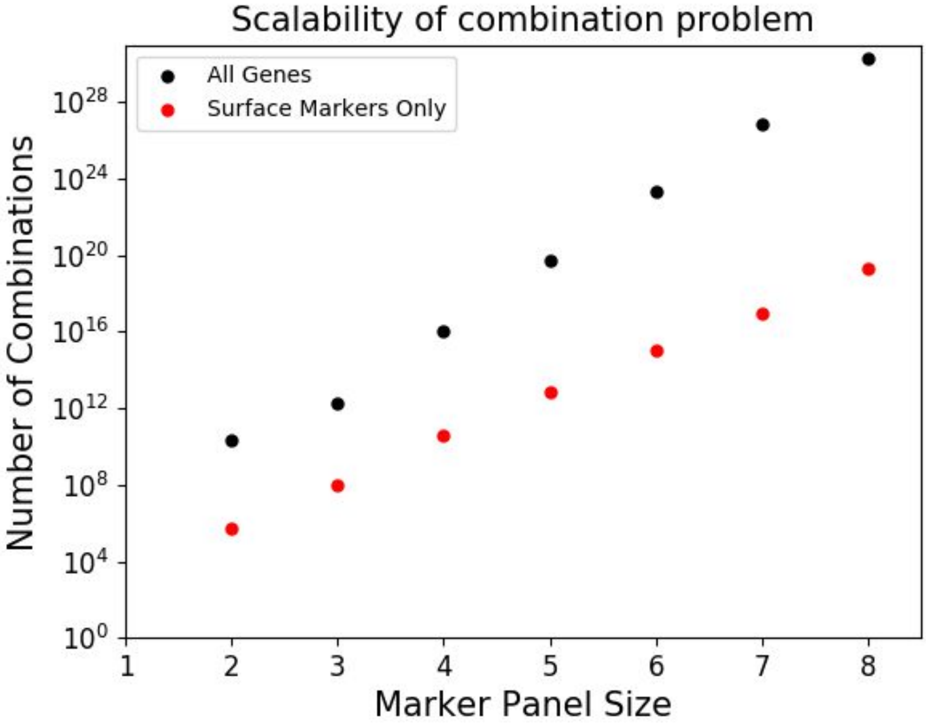
Scalability of combination problem
All Genes
Surface Markers Only
Number of Combinations
Marker Panel Size
10^28
10^24
10^20
10^16
10^12
10^8
10^4
10^0
1
2
3
4
5
6
7
8

Appendix Figure S1. The scale and hardness of identifying multi-gene marker-panels.
A. A hypothetical example showing that a marker-panel achieving optimal accuracy may be composed of genes that are poor markers on their own.
B. The number of different gene-combinations that can be constructed (y-axis) as a function of different sizes of marker panels (x-axis). Plotted are results when selecting marker combinations from the entire mouse gene list (23,433 genes, black) and when gene combinations are selected from a curated list of cell surface markers that is used by default in the COMET framework (979 genes, red) (the gene list to select marker panels from in COMET can be changed by the user). A proof for the computational hardness of identifying optimal marker panels can be found in the Methods section.

Figure S2

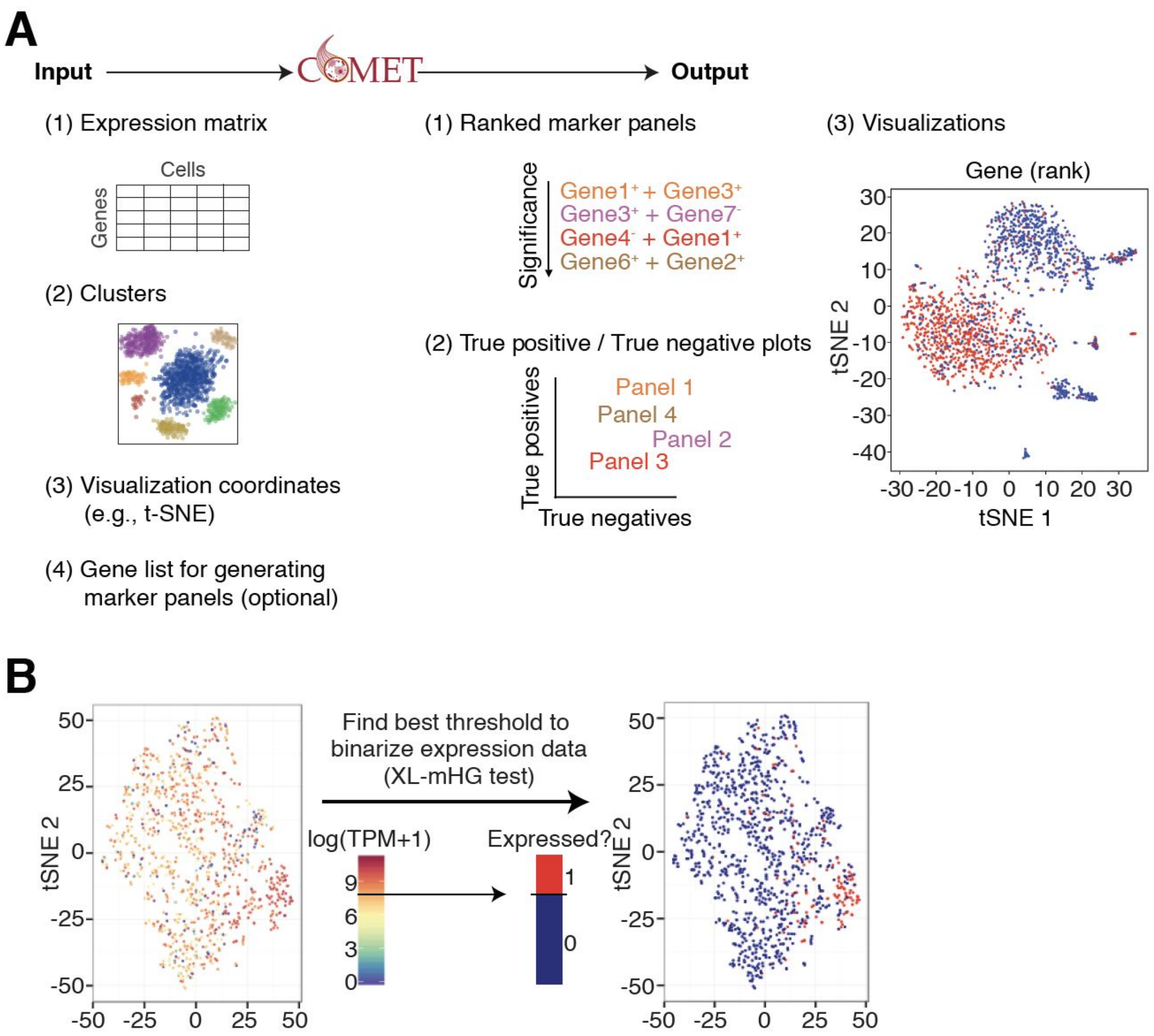

Gene G expression	In Cluster K
21	Yes
16	Yes
15	Yes
13	No
12	Yes
11	Yes
7	No
6	No
3	No
1	Yes
0	No
0	No
0	No

Gene G expression	Binary Gene G expression	In Cluster K
21	1	Yes
16	1	Yes
15	1	Yes
13	1	No
12	1	Yes
11	1	Yes
7	0	No
6	0	No
3	0	No
1	0	Yes
0	0	No
0	0	No
0	0	No

Appendix Figure S2. An overview of the COMET framework including inputs, outputs, and the XL-mHG binarization procedure.
A. Inputs and outputs of the COMET tool, shows what information is necessary to retrieve marker panel results.
B. Extension of Figure 2A, the XL-mHG test sets a threshold of expression and binarizes based on this choice of threshold.

Figure S3

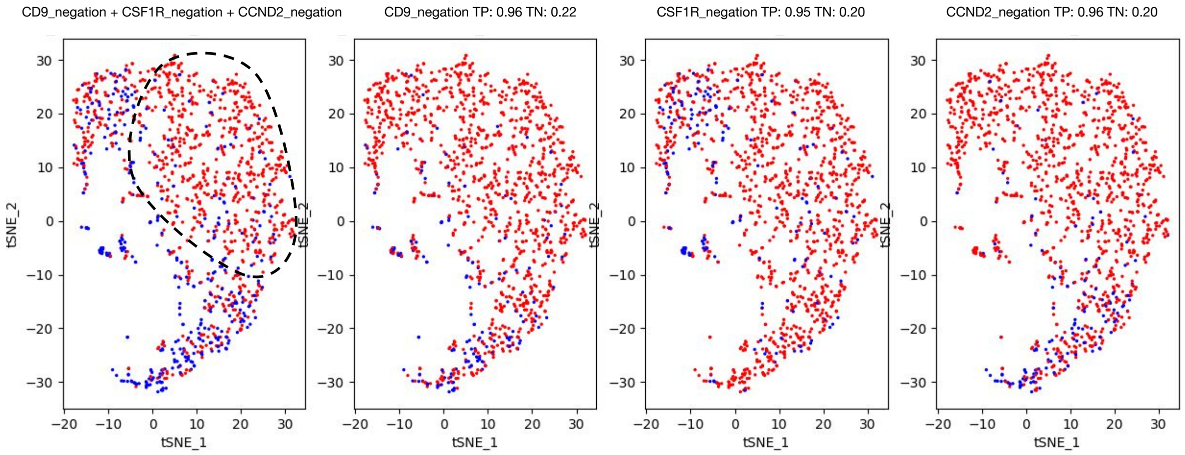
CD9_negation + CSF1R_negation + CCND2_negation
CD9_negation TP: 0.96 TN: 0.22
CSF1R_negation TP: 0.95 TN: 0.20
CCND2_negation TP: 0.96 TN: 0.20
tSNE_1
tSNE_2

Appendix Figure S3. An example of a 3-gene combination output (CD9$^-$CSF1R$^-$CCND2$^-$) from COMET (cluster of interest is circled), generated for the follicular B cell cluster from the splenic data shown in Figure 5A. This example demonstrates how the addition of multiple markers, including negations, can clear out contaminating clusters.

Figure S4

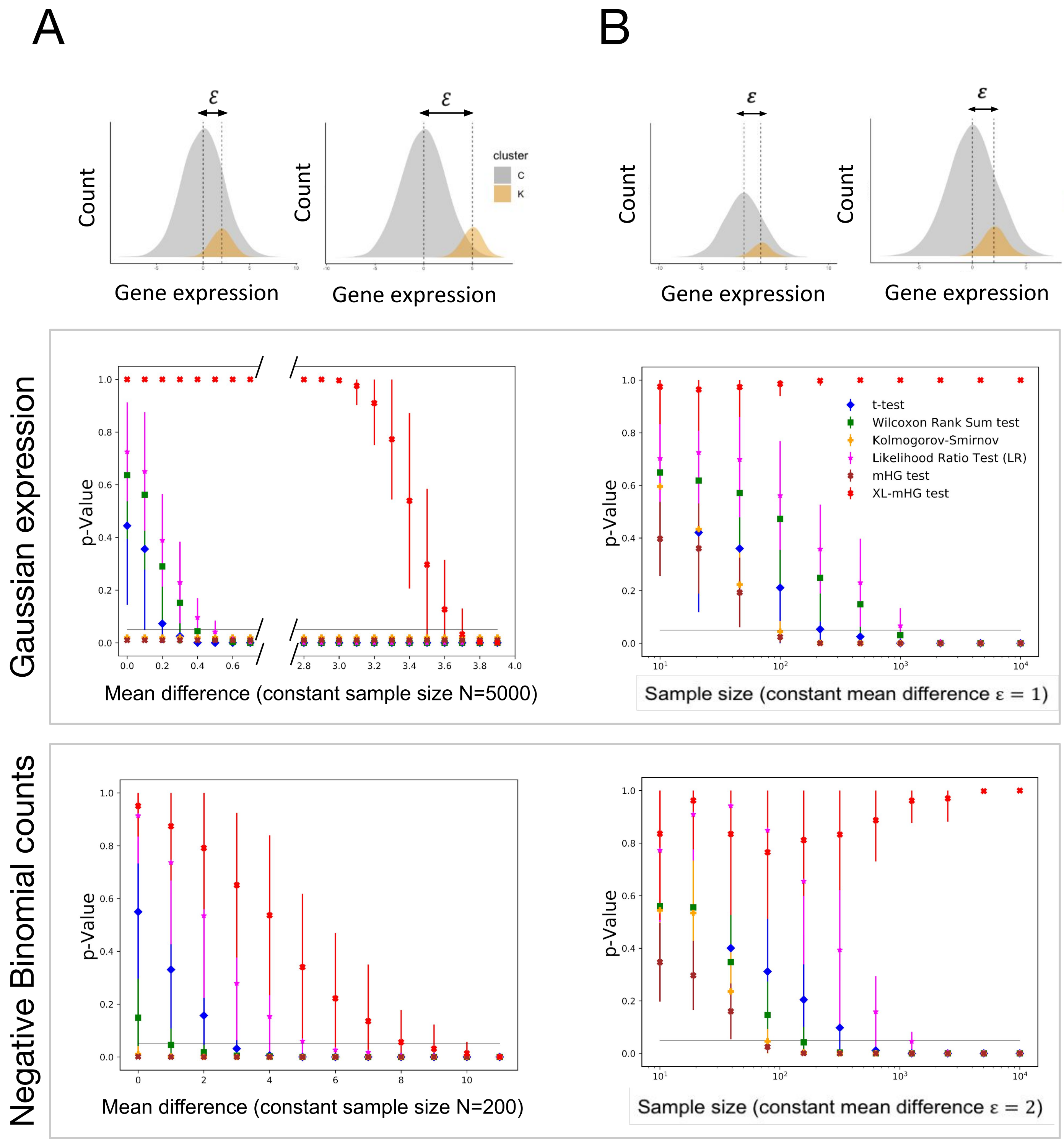
A
B
ε
Count
Gene expression
cluster
C
K
Gaussian expression
Negative Binomial counts
p-Value
Mean difference (constant sample size N=5000)
Sample size (constant mean difference ε = 1)
Mean difference (constant sample size N=200)
Sample size (constant mean difference ε = 2)
t-test
Wilcoxon Rank Sum test
Kolmogorov-Smirnov
Likelihood Ratio Test (LR)
mHG test
XL-mHG test

Appendix Figure S4. The XL-mHG test enjoys desirable properties for marker discovery compared to standard differential expression tests on simulated gene expression data. Simulated data is generated for one gene in two cell clusters and , where denotes the cluster of interest (Methods). Comparison of the XL-mHG test to the standard mHG test reveals the important role of parameters X and L.

A. The XL-mHG test outperforms various differential expression tests in identifying favorable marker genes to be used as markers from simulated datasets (Methods) with respect to robustness to small effect-sizes for Gaussian expression data (center) and Negative Binomial count data (bottom).

B. The XL-mHG test outperforms various differential expression tests in identifying favorable marker genes to be used as markers from simulated datasets (Methods) with respect to sensitivity to sample size for Gaussian expression data (center) and Negative Binomial count data (bottom).

Error bars indicate one standard deviation across 100 simulation runs (thresholded below at 0 and above at 1).

Figure S5

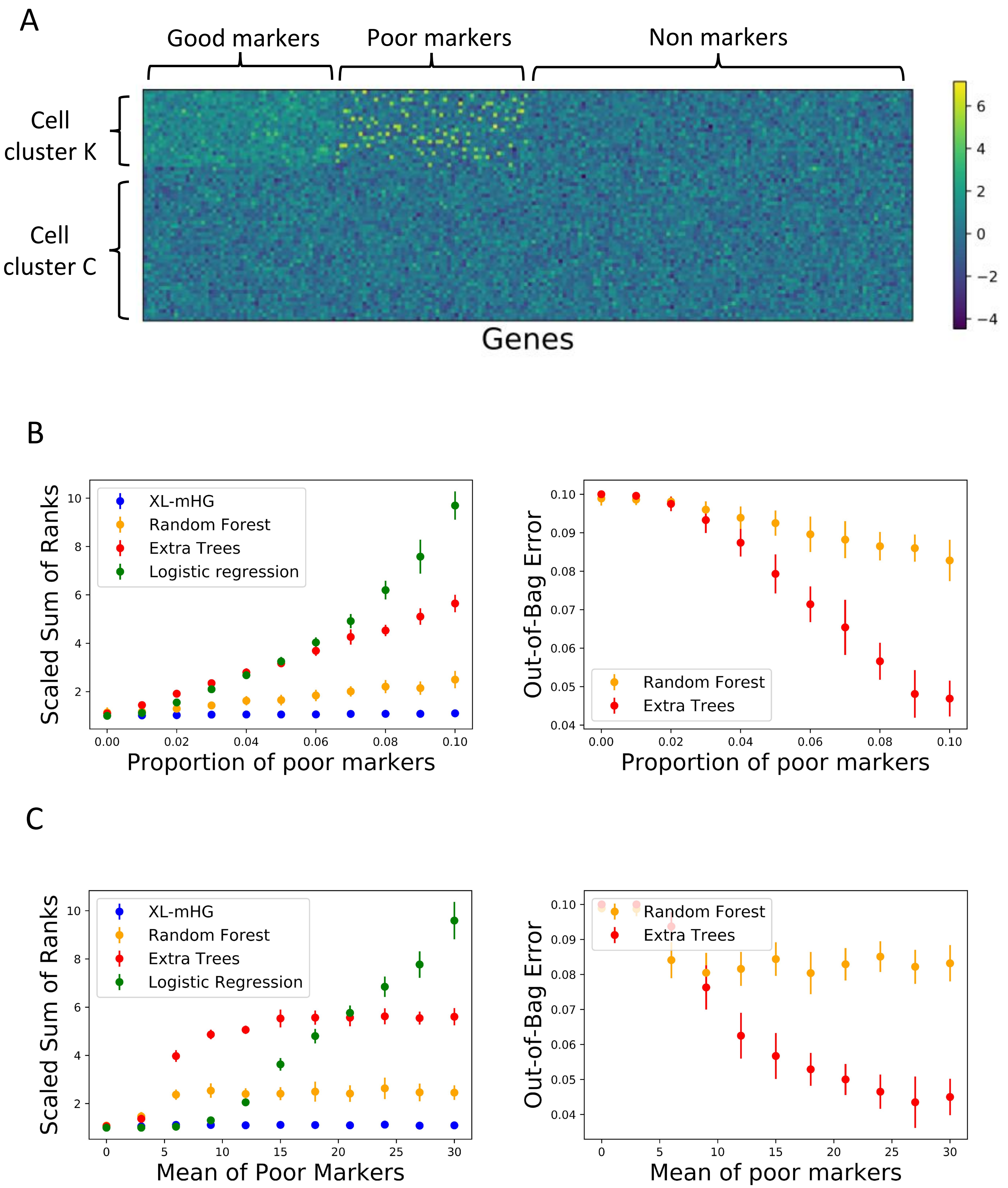
A
Good markers
Poor markers
Non markers
Cell cluster K
Cell cluster C
Genes
6
4
2
0
−2
−4
B
Scaled Sum of Ranks
XL-mHG
Random Forest
Extra Trees
Logistic regression
Proportion of poor markers
Out-of-Bag Error
Random Forest
Extra Trees
Proportion of poor markers
C
Scaled Sum of Ranks
XL-mHG
Random Forest
Extra Trees
Logistic Regression
Mean of Poor Markers
Out-of-Bag Error
Random Forest
Extra Trees
Mean of poor markers

Appendix Figure S5. The XL-mHG test outperforms standard classifiers for marker recovery on simulated Gaussian gene expression data.

A. Outline of the synthetic gene expression matrix generated with *n* cells and *p* genes (Methods). Cells are divided into two clusters and , where denotes the cluster of interest. Three categories of genes are considered: genes which are upregulated in (good markers), genes which are upregulated in only a small subset of cells in (poor markers) and genes which are not differentially expressed across and (non-markers). The Scaled Sum of Ranks (SSR) metric (Methods) is used to assess the performance of four classification methods at recovering good markers from the expression matrix: XL-mHG test, Random Forests (RF), Extra Trees (XT) and Logistic regression. SSR=1 is the optimal value.

B. SSR versus proportion of poor markers in the data set (left). The XL-mHG picks up the correct good markers regardless of the proportion of poor markers, while this proportion affects both LR (via an increase in fold change between and), RF and XT. Out-of-bag error (OOB error) is included for RF and XT (right).

C. SSR versus mean of poor markers in the data set (left). The XL-mHG test picks up the correct good markers regardless of the mean of poor markers. Poor markers with very high expression are valuable for RF and XT, and contribute to increase the fold change between and , resulting in suboptimal performances of RF, XT and LR. Out-of-bag error (OOB error) is included for RF and XT (right).

Error bars indicate one standard deviation across 20 simulation runs.

Figure S6

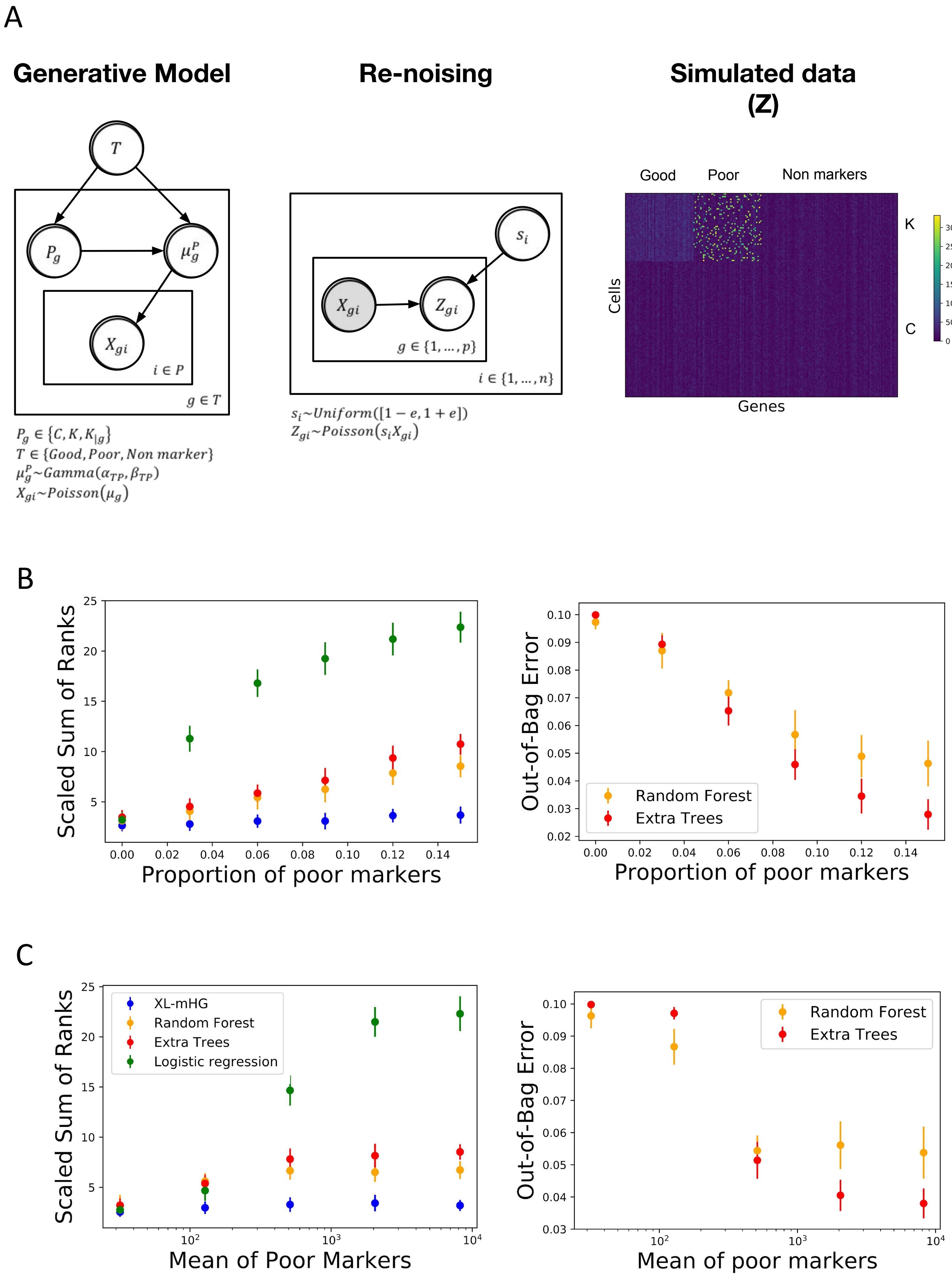

A
Generative Model
Re-noising
Simulated data
(Z)
T
P_g
μ_g^P
X_{gi}
$i \in P$
$g \in T$
s_i
X_{gi}
Z_{gi}
$g \in \{1, \ldots, p\}$
$i \in \{1, \ldots, n\}$
$P_g \in \{C, K, K_{|g}\}$
$T \in \{Good, Poor, Non\ marker\}$
$\mu_g^P \sim Gamma(\alpha_{TP}, \beta_{TP})$
$X_{gi} \sim Poisson(\mu_g)$
$s_i \sim Uniform([1-e, 1+e])$
$Z_{gi} \sim Poisson(s_i X_{gi})$
Good
Poor
Non markers
K
C
Cells
Genes
300
250
200
150
100
50
0
B
Scaled Sum of Ranks
Proportion of poor markers
Out-of-Bag Error
Proportion of poor markers
Random Forest
Extra Trees
C
XL-mHG
Random Forest
Extra Trees
Logistic regression
Scaled Sum of Ranks
Mean of Poor Markers
Out-of-Bag Error
Mean of poor markers
Random Forest
Extra Trees

Appendix Figure S6: The XL-mHG test outperforms standard classifiers for marker recovery on simulated gene counts data.
A. Graphical representations of the simulation engine used to generate synthetic transcriptomic counts data (Methods). Shaded nodes indicate observed variables. A hierarchical Poisson-Gamma model (left) is utilized to generate a cell-by-gene matrix of true counts (ground truth). Technical and efficiency noises are then introduced using an efficiency scaling factor followed by Poisson resampling (center). This procedure produces gene count matrices of the type shown on the right.
B. SSR versus proportion of poor markers in the data set (left). The XL-mHG picks up the correct good markers regardless of the proportion of poor markers, while this proportion affects both LR (via an increase in fold change between and), RF and XT. Out-of-bag error (OOB error) is included for RF and XT (right).
C. SSR versus mean of poor markers in the data set (left). The XL-mHG test picks up the correct good markers regardless of the mean of poor markers. Poor markers with very high expression are valuable for RF and XT, and contribute to increase the fold change between and , resulting in clear suboptimal performances LR. Performances are also suboptimal for RF and XT. Out-of-bag error (OOB error) is included for RF and XT (right).
Error bars indicate one standard deviation across 20 simulation runs.

Figure S7

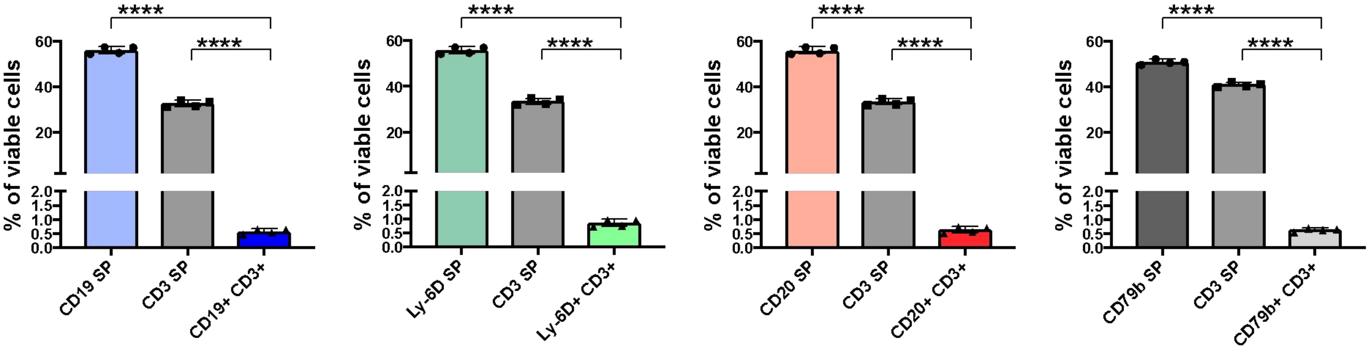

****
****
% of viable cells
60
40
20
2.0
1.5
1.0
0.5
0.0
CD19 SP
CD3 SP
CD19+ CD3+
Ly-6D SP
CD3 SP
Ly-6D+ CD3+
CD20 SP
CD3 SP
CD20+ CD3+
CD79b SP
CD3 SP
CD79b+ CD3+

Appendix Figure S7. Flow cytometry analysis to compare the protein level stainings of CD19, Ly-6D, CD20, and CD79b to the established T cell marker CD3 (Meuer *et al*, 1983). Limited co-staining was observed, highlighting the markers' specificity as B cell markers. (SP= single positive).

Figure S8

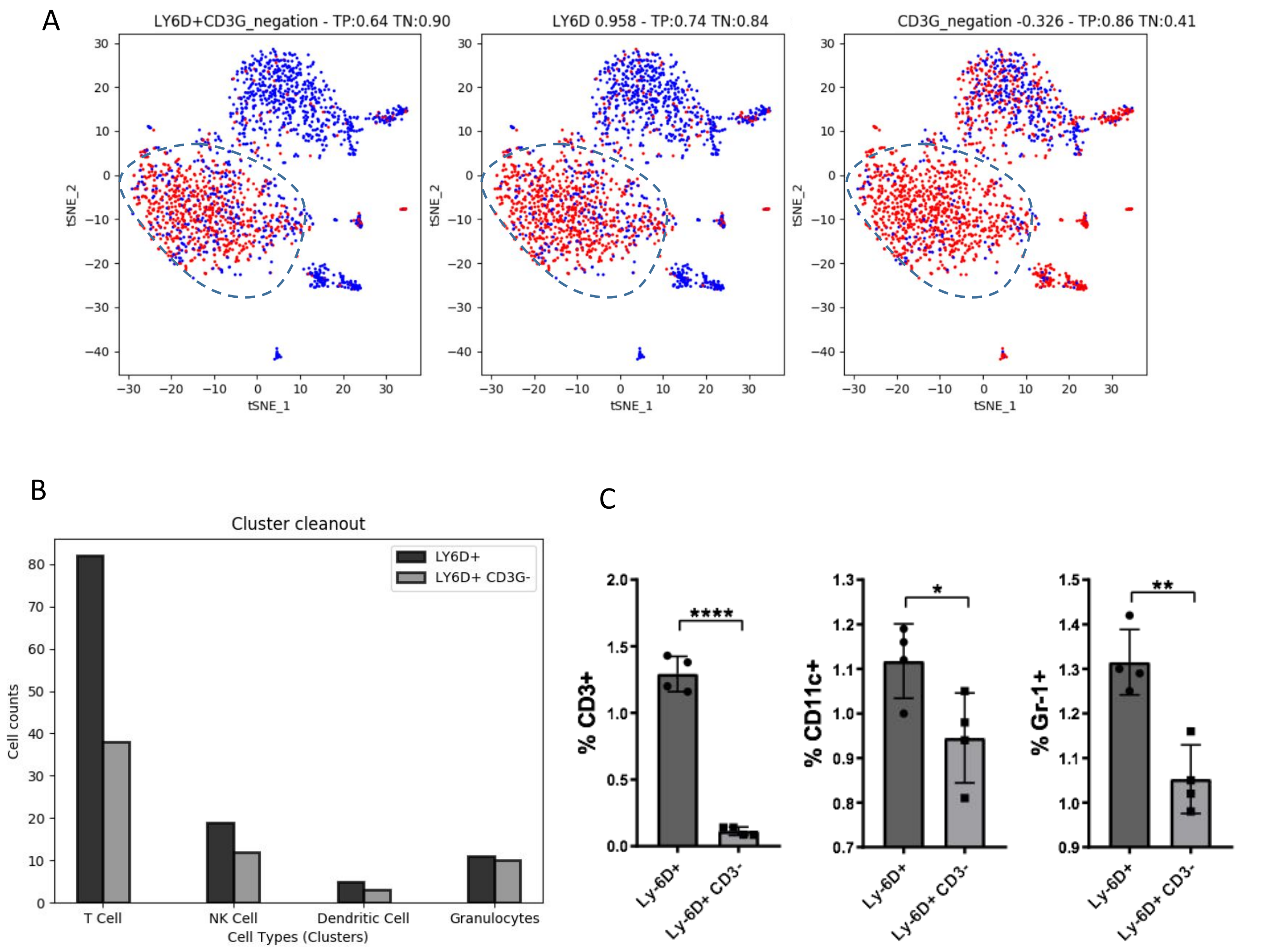
A
LY6D+CD3G_negation - TP:0.64 TN:0.90
LY6D 0.958 - TP:0.74 TN:0.84
CD3G_negation -0.326 - TP:0.86 TN:0.41
tSNE_1
tSNE_2
B
Cluster cleanout
LY6D+
LY6D+ CD3G-
Cell counts
T Cell
NK Cell
Dendritic Cell
Granulocytes
Cell Types (Clusters)
C
% CD3+
% CD11c+
% Gr-1+
****
*
**
Ly-6D+
Ly-6D+ CD3-

Appendix Figure S8. The marker combination Ly-6D$^+$CD3$^-$ predicted by COMET for B cells achieves improved B cell staining.

A. COMET output t-SNE visualization of the marker combination Ly-6D$^+$CD3$^-$.

B. Bar graph showing the overall cell counts in non-B cell clusters for Ly-6D$^+$ and Ly-6D$^+$CD3$^-$, as predicted from the binarization procedure of COMET. Addition of the second marker decreases the overall counts in these other clusters.

C. Flow cytometry analysis of the expression of the T cell marker CD3, the dendritic cell marker (CD11c), and the neutrophil marker (Gr-1) in the Ly-6D$^+$ and Ly-6D$^+$CD3$^-$ populations confirms that the Ly-6D$^+$CD3$^-$ marker panel improves clean out of contamination for all populations tested (Hestdal *et al*, 1991; Merad *et al*, 2013; Meuer *et al*, 1983). The Ly-6D$^+$CD3$^-$ marker panel was selected based on available antibodies.

Error bars indicate the mean and SD. $*p < 0.05$; $**p < 0.01$; $****p < 0.0001$.

Figure S9

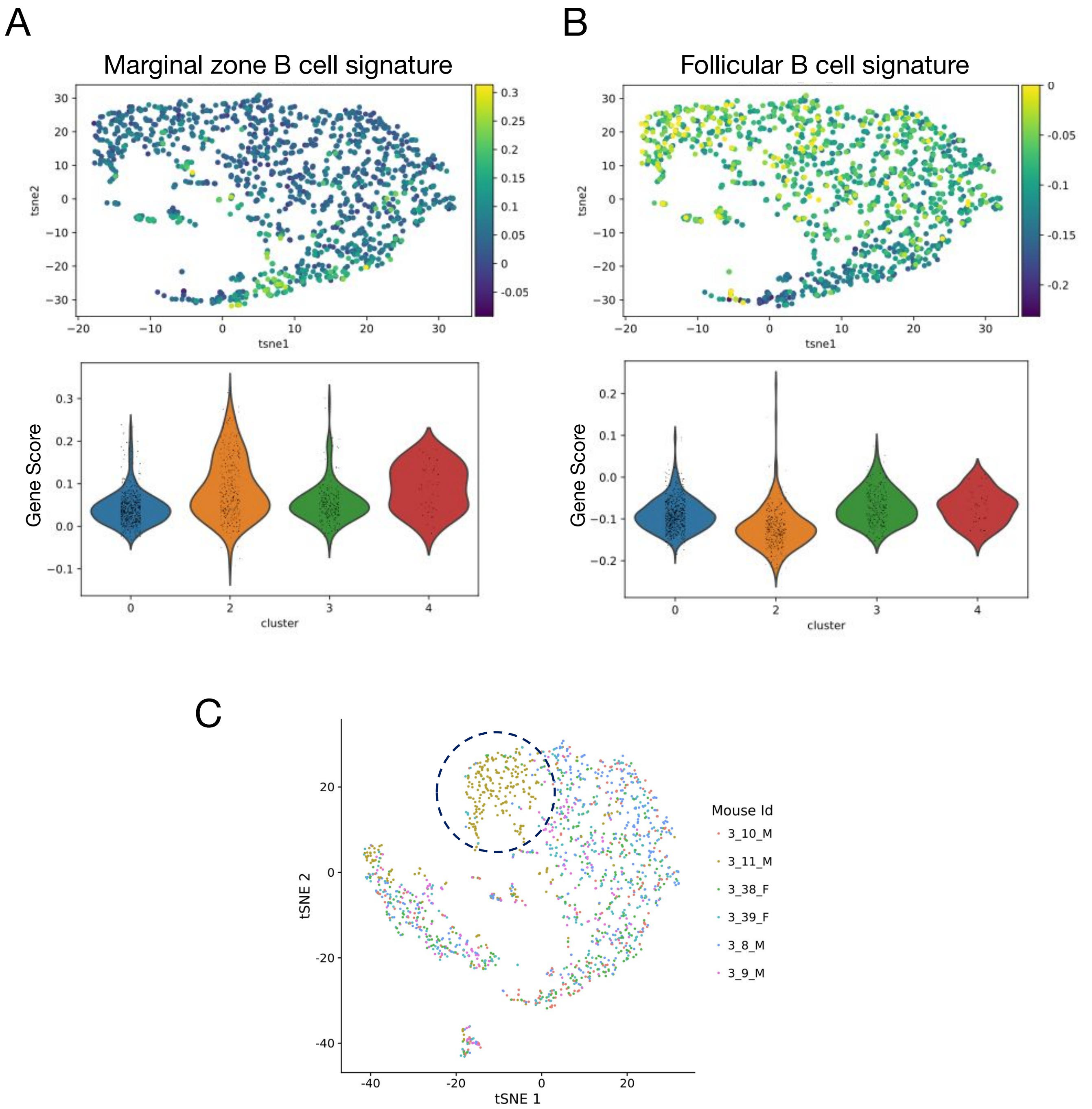
A
B
Marginal zone B cell signature
Follicular B cell signature
tsne1
tsne2
Gene Score
cluster
0
2
3
4
C
Mouse Id
3_10_M
3_11_M
3_38_F
3_39_F
3_8_M
3_9_M
tSNE 1
tSNE 2

Appendix Figure S9. Gene signature scores identify follicular and marginal zone B cell populations.
A. Marginal zone B cell signature scores identify cluster 2 (from Figure 5A) as the marginal B cell cluster (signature: marginal zone B cells vs. follicular B cells (Kleiman *et al*, 2015), Wilcoxon Rank-Sum test *p*-value of 7.8e-25 between clusters 2 and 0). Shown are signature scores computed for each cell (divided into clusters) and violin plots generated by the Scanpy function score_genes. Cluster 4 was not considered marginal due to its lack of expression of the marker gene CD21 (Figure 2C).
B. Follicular B cell signature scores identify cluster 0 (from Figure 5A) as the follicular B cell cluster (signature: follicular B cells vs. marginal zone B cells, Wilcoxon Rank-Sum test *p*-value of 7.5e-31 between clusters 0 and 2) (Kleiman *et al*, 2015). Shown are signature scores computed for each cell (divided into clusters) and violin plots generated by the Scanpy function score_genes. Scores in t-SNE visualization are capped at zero. Cluster 3 was not considered follicular due to a strong batch effect defining that cluster (**C**). Cluster 4 was not considered follicular due to its lack of expression of the marker gene CD23 (Figure 5B).
C. Visualization of splenic immune populations generated by Tabula Muris and available on their website (Tabula Muris Consortium, 2018) website, including all CD45+ cells analyzed. Cluster 3 (circled) in the data contains a significant batch effect driven by mouse of origin. We therefore do not relate to cluster 3 in our follicular / marginal zone B cell analysis.

Figure S10

A

Gene	P-value	Fold change	True positive	True negative	Rank
CD55	1.89E-22	1.21	0.64	0.64	1
SELL	2.46E-21	1.22	0.79	0.47	2
CXCR4	3.98E-08	1.58	0.22	0.89	3
FCER2A	9.18E-21	0.84	0.74	0.50	4
ITGB7	4.84E-05	0.93	0.39	0.73	5
CD2	3.74E-07	0.59	0.55	0.58	6
EPCAM	1.17E-02	1.17	0.21	0.81	7
CCR7	1.05E-08	0.51	0.56	0.60	8
CD200	1.25E-02	0.94	0.29	0.77	9
BTLA	1.33E-03	0.63	0.57	0.54	10

B

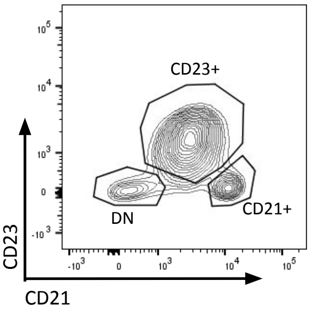

C

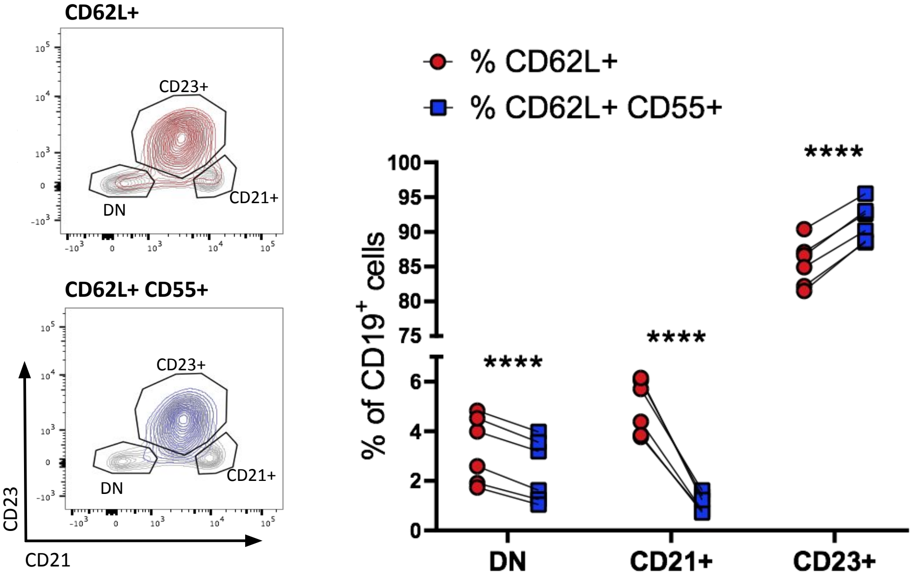

Appendix Figure S10. COMET identifies single- and multi-gene marker panels for splenic follicular B cells.

A. COMET output of the top 10 ranked single genes for the follicular B cell cluster (shown in Figure 5A).

B. Flow cytometry gating strategy for follicular B cells ($CD23^{+}$), marginal zone B cells ($CD21^{+}$), and double negative B cells (DN). All viable $CD19^{+}$ splenocytes were included.

C. Comparison of the marker combination $CD62L^{+}CD55^{+}$ to the single stain $CD62L^{+}$ for the staining of follicular B cells confirms that staining with the 2-gene marker panel improves on staining with CD62L alone. ****$p < 0.0001$.

Figure S11

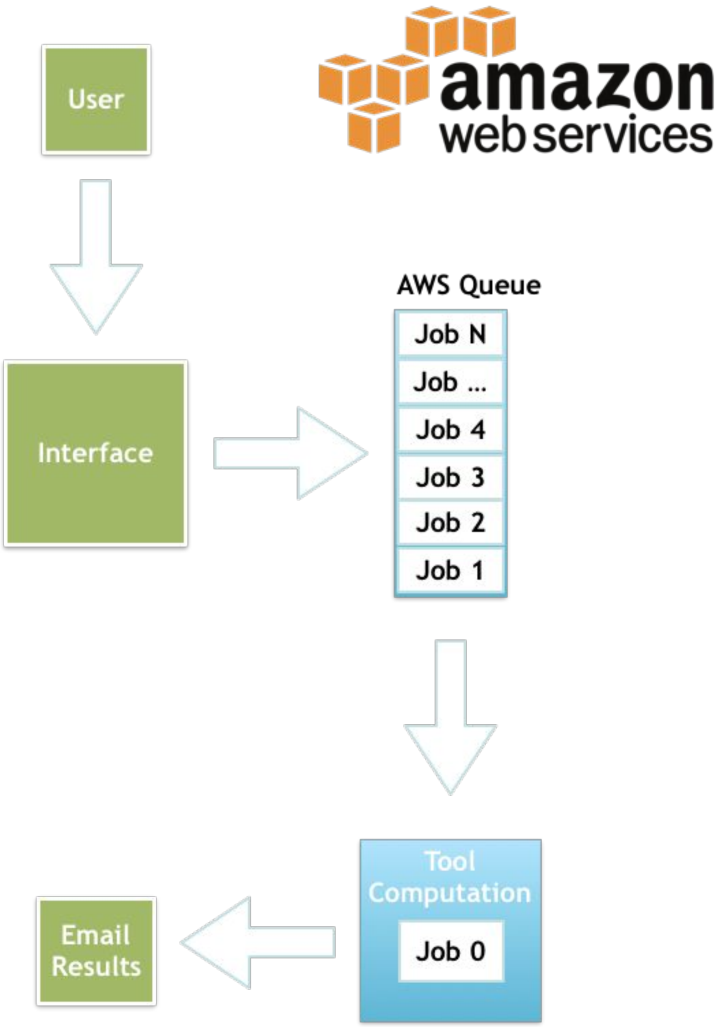
User
amazon
web services
AWS Queue
Job N
Job ...
Job 4
Job 3
Job 2
Job 1
Interface
Tool
Computation
Job 0
Email
Results

Appendix Figure S11. COMET is deployed to users over the web through an Amazon Web Services cloud backend coupled with a Flask application. A queue service (AWS SQS) grabs the submitted jobs and feeds them through a computing instance. Upon completion, a unique job ID is sent to the user's email, allowing them to access the finalized results on any computer. All files are stored in an S3 bucket and available for four days. The basic interface is available at www.cometsc.com and is freely available.

Appendix Reference List

Hestdal K, Ruscetti FW, Ihle JN, Jacobsen SE, Dubois CM, Kopp WC, Longo DL & Keller JR (1991) Characterization and regulation of RB6-8C5 antigen expression on murine bone marrow cells. *The Journal of Immunology* **147:** 22–28

Kleiman E, Salyakina D, De Heusch M, Hoek KL, Llanes JM, Castro I, Wright JA, Clark ES, Dykxhoorn DM & Capobianco E (2015) Distinct transcriptomic features are associated with transitional and mature B-cell populations in the mouse spleen. *Frontiers in immunology* **6:** 30

Merad M, Sathe P, Helft J, Miller J & Mortha A (2013) The dendritic cell lineage: ontogeny and function of dendritic cells and their subsets in the steady state and the inflamed setting. *Annual review of immunology* **31:** 563–604

Meuer SC, Fitzgerald KA, Hussey RE, Hodgdon JC, Schlossman SF & Reinherz EL (1983) Clonotypic structures involved in antigen-specific human T cell function. Relationship to the T3 molecular complex. *Journal of Experimental Medicine* **157:** 705–719

Tabula Muris Consortium (2018) Single-cell transcriptomics of 20 mouse organs creates a Tabula Muris. *Nature* **562:** 367
